# Pacui Virus, Rio Preto da Eva Virus, and Tapirape Virus, Three Distinct Viruses within the Family *Bunyaviridae*

**DOI:** 10.1128/genomeA.00923-14

**Published:** 2014-11-13

**Authors:** Daniela Sueli Guerreiro Rodrigues, Daniele Barbosa de Almeida Medeiros, Sueli Guerreiro Rodrigues, Livia Caricio Martins, Clayton Pereira Silva de Lima, Layanna Freitas de Oliveira, Janaina Mota de Vasconcelos, Daisy Elaine Da Silva, Jedson Ferreira Cardoso, Sandro Patroca da Silva, João Lídio da Silva Gonçalves Vianez-Júnior, Márcio Roberto Teixeira Nunes, Pedro Fernando da Costa Vasconcelos

**Affiliations:** a Department of Arbovirology and Hemorrhagic Fevers, Evandro Chagas Institute, Ministry of Health, Ananindeua, Brazil; b Center for Technological Innovation, Evandro Chagas Institute, Ministry of Health, Ananindeua, Brazil

## Abstract

Nearly complete genome sequences for three ungrouped viruses, Pacui virus (BEAN27326), Rio Preto da Eva virus (BEAR540870), and Tapirape virus (BEAN767592) isolated in the Amazon region are reported here. All three genomic segments (small, medium and large RNA) were recovered and were similar to members of the genus *Orthobunyavirus*.

## GENOME ANNOUNCEMENT

The family *Bunyaviridae* is composed of 350 viruses distributed in five genera (*Hantavirus*, *Nairovirus*, *Orthobunyavirus*, *Phlebovirus*, and *Tospovirus*, which is restricted to plant infection) (1) and some unclassified species (2). In addition, a potential prototype of a sixth genus was described, Gouleako virus, which was related to phleboviruses (3).

A large number of bunyaviruses are associated with human and productive animal disease and are important health and veterinary threats. Members of the family *Bunyaviridae* are vector-borne viruses (mosquitoes, ticks, sand flies, and thrips), with the exception of hantaviruses, which are basically transmitted by contact with excretes of wild rodents (feces and urine) (4). These viruses are spherical (80- to 120-nm diameters) and possesses a tripartite single-stranded negative genome (small RNA [SRNA], medium RNA [MRNA], and large RNA [LRNA]). In general, six proteins are encoded: three structural proteins N, Gn, and Gc glycoproteins, as well as three nonstructural proteins (NSs, NSm, and viral polymerase). Noncoding regions (NCR) are present at the 5′ and 3′ termini of each genomic RNA segment (1).

Pacui virus (PACV) was isolated from a rodent *Oryzomys* sp. in the Belém-Brasilia highway, Pará State (03°S, 48°W), Brazil, in 1961. Rio Preto da Eva virus (RPEV) was isolated from a pool of *Psychodidae* sp. (*Diptera*: *Phlebotominae*) in the Rio Preto da Eva municipality (2°41′55″S; 59°42′3″W), Amazonas state, Brazil, in 1995. Tapirape virus (TPPV) was isolated from an *Oxymycterus* sp. rodent captured in the Zoobotanic Park area, Parauapebas municipality, Pará State, Brazil (6°3′46″S; 50°3′33″W).

Viruses were cultivated into Vero cells, and after 90% cytopathic effect (CPE) they were harvested and their genomes recovered after RNA extraction using the Trizol/Quiagen method as recommended by the manufactures. Nearly complete genomes were obtained using the GS FLX 454 (Roche, Life Science) sequencing.

The genomes were assembled by use of a *de novo* strategy (5) and the GS *de novo* Assembler program implemented in the Newbler v. 6 software. Visual inspection, open reading frames (ORFs), and gene predictions were performed with the software Geneious v. 7. For PACV, the total genome recovered was 12,467 nucleotides (nt) in length (S, 948 nt; M, 4,648 nt; L, 6,871 nt); for RPEV, the genome was 12,329 nt (S, 938 nt; M, 4,579 nt; L, 6,812 nt); and for TTPV, the genome was 12,234 nt in length (S, 949 nt; M, 4,446; L, 6839 nt). All three viruses showed the N, Gn, Gc, NSm, and polymerase genes, but the NSs gene was not found. This is the first report of the complete ORF genome sequences for PACV, RPEV, and TPPV, three ungrouped bunyaviruses isolated in Brazil.

### Nucleotide sequence accession numbers.

The genome sequences have been deposited in GenBank under the following accession numbers: PACV (S, KM225256; M, KM225255; L, KM225254); RPEV (S, KM225259; M, KM225258; L, KM225257); and TPPV (S, KM225262; M, KM225261; L, KM225260).
